# Transformer-based deep learning for predicting protein properties in the life sciences

**DOI:** 10.7554/eLife.82819

**Published:** 2023-01-18

**Authors:** Abel Chandra, Laura Tünnermann, Tommy Löfstedt, Regina Gratz

**Affiliations:** 1 Department of Computing Science, Umeå University Umeå Sweden; 2 Umeå Plant Science Centre (UPSC), Department of Forest Genetics and Plant Physiology, Swedish University of Agricultural Sciences Umeå Sweden; 3 Department of Forest Ecology and Management, Swedish University of Agricultural Sciences Umeå Sweden; Goethe University Germany; Goethe University Germany

**Keywords:** deep learning, transformers, life sciences, protein property prediction, machine learning

## Abstract

Recent developments in deep learning, coupled with an increasing number of sequenced proteins, have led to a breakthrough in life science applications, in particular in protein property prediction. There is hope that deep learning can close the gap between the number of sequenced proteins and proteins with known properties based on lab experiments. Language models from the field of natural language processing have gained popularity for protein property predictions and have led to a new computational revolution in biology, where old prediction results are being improved regularly. Such models can learn useful multipurpose representations of proteins from large open repositories of protein sequences and can be used, for instance, to predict protein properties. The field of natural language processing is growing quickly because of developments in a class of models based on a particular model—the Transformer model. We review recent developments and the use of large-scale Transformer models in applications for predicting protein characteristics and how such models can be used to predict, for example, post-translational modifications. We review shortcomings of other deep learning models and explain how the Transformer models have quickly proven to be a very promising way to unravel information hidden in the sequences of amino acids.

## Computational protein property prediction

Proteins have properties that could either be global or local, that is, wholistic protein properties (e.g. stability of a protein) or regional protein properties (e.g. phosphorylation of an amino acid residue by a protein kinase). These different protein properties are usually determined through wet lab experiments, which can be challenging, time-consuming, and costly. The change in protein stability based on changes in protein sequence, for example, requires measuring the change in Gibbs free energy of folding of the purified wild-type and mutant proteins (Walls and Loughran, 2017). Even though this experimental procedure provides direct understanding of protein stability, much time and high costs are involved, especially when multiple mutations in a sequence need to be analysed. This has driven interest into computational methods to guide mutation analysis and design (Pan et al., 2022). Employing a computational approach can also aid the experimental approach by providing a ranked list of predictions for a property (e.g. to predict the likelihood of interaction between two given protein sequences) that can be experimentally verified or refuted by scientists in focused experimental testing, which can save much time and other resources (Ehrenberger et al., 2015).

There has been an exponential growth in the number of protein sequences collected in public repositories using high-throughput technologies. However, the gap between the number of sequenced proteins and the number of protein property annotations continues to widen (UniProt Consortium, 2019; Varadi et al., 2022). Recently, machine learning (ML) methods, in general, and large-scale deep learning (DL) methods, in particular, have gained much attention due to their ability to extract complex patterns from large collections of protein data (Shi et al., 2021; Li et al., 2022) to automatically predict protein properties. There is now a vast and growing number of applications of DL methods used in the proteomic field that assist in building knowledge about various protein properties.

Since recent large-scale DL models have played a crucial role in computational protein property prediction (Bileschi et al., 2022), we describe in this review the most common DL architectures in use today. DL methods, especially those coming from the field of natural language processing (NLP), are gaining popularity, and we therefore discuss DL methods in the context of NLP. We denote such models as *language models*. Further, we explain how language models relate and have been adopted to analyse protein sequences. Various language models have been developed in the protein area, and we highlight some recent examples where they have been used to predict protein properties in the life sciences. In particular, we discuss and explain the Transformer model, which has managed to overcome several of the shortcomings of previous methods. We further provide a proof-of-principle example, where we predict a post-translational modification (PTM) in proteins. PTMs are a common way of changing a protein’s functionality and are often associated with regulatory cascades and cellular signaling. PTMs can be determined in wet lab settings, for example, with mass spectrometry, but can also be predicted using computational approaches. In our proof-of-principle example, we set out to predict whether lysine residues in proteins are phosphoglycerylated or not. To do this, we compared the prediction performance when using traditional protein features, determined by the analyst, to the performance when using features automatically found using two types of Transformer models. By feature here, we mean, for instance, some description of a protein, a statistic, or a measurement. Finally, we discuss the future of protein property prediction and predict that Transformer-like models will be the standard approach for many computational biology and bioinformatics tasks in the near future.

## A brief introduction to deep learning

ML is a subarea of artificial intelligence (AI), and much of the recent developments within the field of AI come from progress made within ML. The aim of ML is to use data to solve a task, for instance, to predict a specific protein property based on measurements, that is*,* data, from other proteins where those properties are known. Most of the recent ML developments have been made within DL, a subarea of ML. However, NLP is also a subfield of AI, where the aim is to use computers to analyse and understand natural language, which is naturally evolved human language, and often uses ML to process and analyse text data. There is an overlap between ML/DL and NLP, however, and ideas flow both ways between the fields. Recently, developments in NLP have been driving much of the development within all of ML and DL.

**Figure 1.**
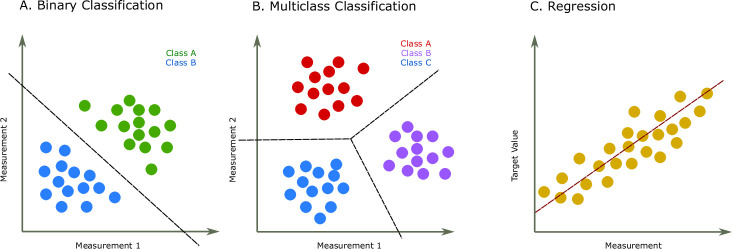
Two common prediction tasks in machine learning (ML) are classification and regression. For illustration purpose, two-dimensional plots are used, but in reality, the dimensions are much higher. (**A**) Binary classification tasks are for samples that can be separated into two groups, called classes. For instance, the samples can be several features of some proteins, where each protein is associated with one of two classes. A protein variant could either be stable or unstable (Fang, 2020) or a lysine residue could be phosphoglycerylated or non-phosphoglycerylated (Chandra et al., 2020). The ML task would be to build a model that can determine the class for a new sample. (**B**) The multiclass classification task is performed when the proteins belong to one of multiple classes. For instance, predicting which structural class a protein belongs to Chou and Zhang, 1995. (**C**) The regression task is for applications where we want to predict real output values, for example, the brightness of a fluorescent protein (Lu et al., 2021).

DL methods are often based on deep artificial neural network models, a class of ML models that are very flexible in the sense that they are able to model very complicated relationships between the measurements (the input data, such as amino acid sequences) and the quantities to be predicted (such as a protein property). The main advantage of neural network models is that they can automatically learn rich feature representations, and they do that directly from large unstructured input data. This means, for instance, that they can take variable-length protein sequences as inputs and automatically find a way to represent them as a fixed-length real (floating-point) vector, where the learned representations contain all the relevant information from the protein sequences that is necessary to solve a particular prediction problem. Having found such a representation, these models also automatically perform a traditional machine learning tasks in the newly learnt representation, such as classification or regression (Charte et al., 2019). Some examples of machine learning tasks are illustrated in Figure 1. In contrast, traditional ML models typically rely on input features determined by the analyst, which are computed from the raw unstructured data, after which a model is determined using those features in a second step. DL models are instead end-to-end systems, meaning that there is only a single step where they automatically learn to map directly from the input data, through an internal learned representation, the automatically determined features, to the target quantities that we want to predict, and they do this with unprecedented accuracy (Khan et al., 2019).

The *architecture* of a neural network model is the network layout and its components, such as the number of artificial neurons in each layer (the number of computational units in the layer), the number of layers (the number of levels of abstractions it can learn), and the type of connections between these layers (which layers are connected to which). The architecture of the network governs the overall behaviour of the neural network, what it can learn, and what assumptions about the data are built in.

**Figure 2.**
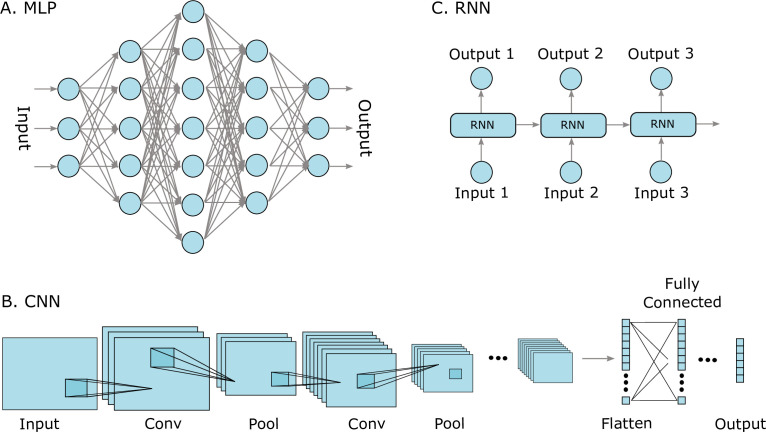
Three well-known deep learning models. (**A**) Multilayer perceptrons (MLPs) are characterized by an input layer, several hidden layers, and an output layer. (**B**) Convolutional neural networks (CNNs) use convolution operations in their layers and learn filters that automatically extract features from the input sequences (e.g. from images, audio signals, time series, or protein sequences). At some point, the learned image features are strung out as a vector, called flattening, and are often passed on to fully connected layers at the end. (**C**) A recurrent neural network (RNNs) is a model that processes an input sequence step-by-step with one element in the sequence at a time.

There are many types of neural network models that are made for analysing different types of data. Some of the most well-known and successful types of neural network models include multilayer perceptrons (MLPs), convolutional neural networks (CNNs), and recurrent neural networks (RNNs) (Koumakis, 2020; see illustrations in Figure 2). These models have been used by themselves, as well as in combination as hybrid models. Examples include work on protein fold recognition, which used a CNN for feature extraction together with an RNN model (Liu et al., 2020), and the popularly used Word2Vec model that can provide embeddings for words to be used in language processing by neural networks, as in the continuous bag-of-words model and the continuous skip-gram model (Mikolov et al., 2013a). MLPs are characterized by an input layer that accepts any type of inputs, for instance, features computed from proteins, several possible interconnected so-called hidden layers, and an output layer with the predicted value, such as a protein property. CNNs use convolution operations, or, technically, they use what is called linear spatial operation, in at least one of their layers. The convolution layers learn filters that detect features, or patterns, in the input signals. The filters automatically capture features in a local region, called the receptive field, and in order to capture feature more distantly, CNNs learn a hierarchy of features. The pooling operation after the convolution layer reduces the dimensionality of the captured features. CNNs have been very successful when analysing image and audio input data, and are very common in computer vision for analysing images, but have also been used to analyse protein sequence data (for instance, DNA-protein binding; Zeng et al., 2016). RNN models have gained much attention since they were first introduced (Elman, 1990) and have been applied widely in many NLP tasks, such as speech recognition (Mikolov et al., 2011). They are suitable for modelling sequential data such as text or time series data, but can also model DNA and protein sequences. The elements in the sequence, for example, words or amino acids, are processed step-by-step one at a time using so-called recurrent connection units, where the output of each step depends on both the current and the previous steps. Common RNN models include the long short-term memory (LSTM) model (Hochreiter and Schmidhuber, 1997) and the gated recurrent units (GRUs) (Chung et al., 2014). There are also models that learn both forwards and backwards, such as the bidirectional LSTM model, BiLSTM (Huang et al., 2015), which uses two LSTM models to capture information from a sequence in both directions. RNNs can model the contextual dependencies in language and were preferred over MLPs and CNNs for most NLP tasks for a long time (Yin et al., 2017).

## Basics of natural language processing

The field of NLP was founded in the 1950s and today covers a wide range of applications, such as sentiment analysis (extracting subjective qualities, like emotions, from text), named entity recognition (classify named entities from text, such as places or person names), machine translation, or question answering. Early NLP systems comprised hand-coded and rule-based assessments (grammar rules) by humans that were then encoded into special-purpose algorithms to predict some property of the sentence. This, however, produced unsatisfactory results and generally failed to deliver when applied to larger text volumes. More recent NLP systems often utilize DL models to automatically learn to solve natural language tasks based on very large volumes of raw, unstructured, and unlabelled text datasets.

**Figure 3.**
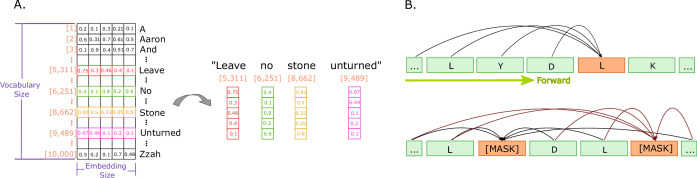
Illustrations of embeddings and of next and masked token predictions. (**A**) An illustration of real-valued vector representations (input embeddings) of the tokens for a sample sentence. Each square represents a numerical value in the vector representation. The vector for each word in the sentence is obtained by looking up the unique ID attributed to the word with the ID in a vocabulary. Each word embedding is of the same size, called the embedding size, and they must be found in the vocabulary (in the illustration, the vocabulary size is 10,000 words). (**B**) The two main training approaches for protein language models, and specifically for Transformers. The top part illustrates autoregressive language modelling (predicting the next token), and the bottom part illustrates masked language modelling (predict a few missing, or masked tokens).

To perform an NLP task, the input text data must be pre-processed to be in a form suitable for automation. The first steps in this process involve splitting the text up into either sentences, words, or parts of words. This process includes a step called tokenization, and the units the text is broken down into are called tokens. These tokens are translated into numerical representations called input embeddings, such as one-hot encoding, count vectors, or word embeddings (Wang et al., 2018), as illustrated in Figure 3A. Word embeddings are the most common real-valued vector representations of the tokens (Levy and Goldberg, 2014) and are often automatically learned (Turian and Ratinov, 2010).

The ability of DL methods to automatically learn feature representations of the input data significantly reduces the need for manual specification or extraction of features by natural language experts. Meaningful information can be extracted from unstructured data using DL methods at a fraction of the time and cost and also often considerably better than human experts (Young et al., 2018; Nauman et al., 2019). The most recent NLP methods, based on a particular model called the Transformer, learn feature representations automatically through the process of unsupervised learning, often called self-supervised learning (Devlin et al., 2018; Peters et al., 2018).

Most NLP tasks today are solved using DL methods based on the Transformer, with existing methods constantly being improved and new methods proposed (Raffel et al., 2019; Brown et al., 2020; Heinzinger et al., 2021). Recent models are also trained on text data of ever-increasing sizes, which have made them perform even better.

## The Transformer model

**Figure 4.**
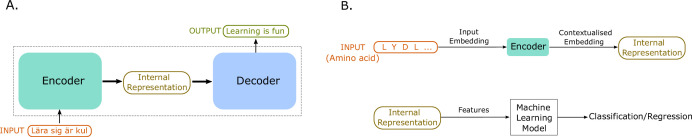
An illustration of sequence-to-sequence models and of how to use the internal representations for down-stream machine learning tasks. (**A**) The conceptual idea behind sequence-to-sequence models. The Transformer model by Vaswani et al., 2017 has a similar form, to map the input sequence to an output sequence using an encoder and a decoder. (**B**) An example application of the Transformer language model for protein property prediction. The input embedding is contextualized using the encoder block, which gives an internal representation, the model’s embedding of the input sequence. The internal representation is then used as features of the amino acids and can be passed in a second step to a machine learning model. The decoder block is not normally used after training since it does not serve much purpose in protein property prediction but is a critical component for training in natural language processing (NLP) applications such as language translation.

The Transformer model was introduced in 2017 by Vaswani et al., 2017 and achieved state-of-the-art results in language translation using only a fraction of the previous training times. It is an encoder–decoder type of model (see Figure 4A for the general idea of an encoder–decoder model), where the encoder maps the vector representations of the tokens from an input text (the input embeddings) to an internal representation. The decoder then uses the internal representation and maps it to the output sequences (the target language, for instance). Compared to contemporary models at the time, the Transformer model did not use recurrent layers, nor did it use convolution layers—instead, it used an architecture component called *attention*. The attention module enables a model to consider the interactions among every pair of tokens in a sequence and automatically learn the relationships between tokens in a sequence that are relevant for the task at hand (Cheng et al., 2021). There are many kinds of attention modules, but most of them, and the one used by Vaswani et al., automatically learn an interaction pattern between pairs of tokens. These interaction patterns give an importance weight to each input token for the prediction task at hand and allows the model to learn dependencies between tokens far apart in the input sequence. In most cases, not just one but several such attention modules are used in parallel, allowing the model to learn multiple different aspects of the relationships between input tokens—this is called multihead attention.

The Transformer model by Vaswani et al. comprises multihead attention (eight parallel attention heads) and fully connected feed-forward networks (these networks are in fact MLP models, used as intermediate components in the overall Transformer model) in six layers of both the encoder and decoder blocks. To generate the input embeddings to the model, the authors used two schemes: encoding the sentences using byte-pair encoding (Britz et al., 2017) or splitting tokens into word-piece vocabulary (Wu et al., 2016), based on the training dataset. The model’s embedding layers produce contextualized embeddings (the internal representations) of size 512 (per token). The multihead attention in the different layers of each block of the network enabled the model to learn rich and useful representation by considering information from tokens at different positions in the input sequence.

The procedure used to train such models is called self-supervision and is typically one of the following two approaches: (1) to predict the next token in a sequence, given the previous tokens (Peters et al., 2018) (this is called autoregressive language modelling), or (2) predict 'masked' tokens, where certain tokens are removed (typically 15% in an input sequence), and the model is made to predict them using the information available in the unmasked tokens in the sequence (this is called masked language modelling [MLM]) (Devlin et al., 2018). These two types of training approaches are illustrated in Figure 3B. The approach originally employed, already before the Transformer models, was to predict the next token in the input sequence. Transformers trained using the MLM approach have become very popular and successful, likely because it allows the model to consider the whole input sequence directly instead of everything up until the present point in the sequence (Nambiar et al., 2020; Elnaggar et al., 2020a; Rives et al., 2021; Brandes et al., 2021; Rao et al., 2021; He et al., 2021).

Many other models, based on the Transformer model, have been proposed after the introduction of the original Transformer. These models all have attention modules as their core components. An example is the BERT model (Devlin et al., 2018), which is an encoder model that has achieved outstanding performance compared with other language models on many NLP tasks, such as machine translation, question answering, etc. (Chung et al., 2014; Cheng et al., 2021). The BERT model attained new state-of-the-art results on 11 NLP tasks. The work demonstrated that bidirectional pre-training is important for language representations and that the pre-trained model can be adapted to many other specific tasks, which is relatively inexpensive compared to building separate models for each individual task. There were two primary models developed: BERT_BASE_ (with 12 layers, 12 attention heads, and 768-dimensional contextual embeddings) and BERT_LARGE_ (24 layers, 16 attention heads, and 1024-dimensional contextual embeddings). The authors found that the BERT_LARGE_ results surpassed the results of BERT_BASE_ on all the tasks, which indicates the importance of the model size for the performance.

Because of the recent successes of DL-based language models in a myriad of NLP tasks, and particularly so when using the Transformer model, there has been an increased interest in such models for applications in other fields, such as in computational biology and bioinformatics. In these fields, NLP models can be applied to sequences of, for example, genomic or proteomic data, and recent results indicate that this is a highly successful approach for protein prediction applications (Choromanski et al., 2020).

DL models are known to be computationally expensive and to take considerable amount of time to train. The Transformer models, however, avoid some of the challenges associated with traditional DL methods for sequence modelling.

For instance, RNN models capture information from previous positions in an input sequence, advancing from the beginning of the sequence (forward). But in doing so, they do not capture any input sequence context from the other side of the current position in the sequence. They also suffer from some fundamental problems (called the vanishing and exploding gradient problems) (Bengio et al., 1994; Pascanu et al., 2013; Hanin, 2018), which makes them difficult to train (Dai et al., 2018). The effect of this is that RNN models have problems to learn relationships between distant tokens in an input sequence (Bengio et al., 1994; Pascanu et al., 2013; Hanin, 2018). Also, since the data is processed one token at a time, it is not possible to parallelize the computations, making the training slow (Wang et al., 2019).

For a CNN to capture distant features in the input, they need to learn a hierarchy of features. It may take many such hierarchy levels to extract meaningful information from a larger part of an input sequence (Raghu et al., 2021), which can make CNNs slow. CNNs are also invariant to spatial translations and do therefore not utilize the positional information that may be relevant in an input sequence (Albawi et al., 2017). While CNNs have had a remarkable success on, for example., image data, they have not been as successful in sequence modelling.

The Transformer models solve many of the hurdles faced by conventional DL approaches, some of which were described above. The Transformer model’s attention module allows each token to influence weights for every other token in the sequence. This allows the Transformer model to attend to long-range dependencies between input tokens, a very beneficial property since it enables Transformers to consider the whole context of an input sequence (Dehghani et al., 2018). As a result, they obtain superior results and sequence embeddings (Väth et al., 2022). The direct connections between distant tokens also help when training the Transformer models, making it easy to train them (Dai et al., 2018). The Transformer models are also highly parallelizable, and only have simple components such as attention modules and fully connected layers, which makes them computationally attractive (Wang et al., 2019).

## Protein language models and representation learning

The models used in the field of NLP can thus also be used to learn and understand protein sequences and in this context, they are commonly referred to as *protein language models* (Heinzinger et al., 2019). While there are abstract similarities between sentences and protein sequences, there are of course major differences in their properties, syntax, and semantics (Ofer et al., 2021). When handling proteins, a word can be one of the individual twenty canonical amino acids (excluding unconventional and rare amino acids) Lopez and Mohiuddin, 2020 found in the genetic code or it could be a number of these amino acids grouped together, while a protein sequence would correspond to a sentence (Ferruz et al., 2022; ElAbd et al., 2020). A word being individual amino acids is the most common approach, and other alternatives do not appear to have been explored much (Ofer et al., 2021). Just like with natural language, protein sequences contain long-range dependencies, making them excellent candidates for analysis by recent NLP models such as Transformers (Ofer et al., 2021).

Figure 4B illustrates how a Transformer language model can be applied to protein sequences. The encoder maps the amino acid tokens of an input protein sequence to an internal representation (the model’s embedding of the protein sequence). This internal representation is then used as a feature vector that represents the protein sequence and is passed on to a conventional machine learning model for classification or regression, for instance. For clarity, we will denote this internal representation the *representation* of a protein sequence in a given protein language model.

For properties of proteins, such as their 3D structure, the mapping from a sequence of amino acids to the corresponding 3D structure is quite challenging (Kuhlman and Bradley, 2019; Jiang et al., 2017), but there is typically an abundance of sequenced proteins openly available that a DL model can make use of. The largest open sources of protein sequence information and data are the Universal Protein Resource (UniProt) (UniProt, 2021), Pfam (Mistry et al., 2021), and the Big Fantastic Database (BFD) (BFD, 2022). UniProt contains around 0.567M sequences that are reviewed and manually annotated and more than 230M sequences that are automatically annotated. The Pfam is a database containing protein families, where a family is determined by similar functional domains. Pfam currently has 19,632 families and clans (higher-level groupings), as per Pfam 35.0, and contains a total of 61M sequences (Pfam 35.0, 2021). BFD is a very large collection of protein families publicly available. It comprises 65.9M families covering more than 2B protein sequences and was built using UniProt, and a reference protein catalogue (Steinegger et al., 2019), clustered to 30% sequence identity. Protein language models are typically trained on such large open collections of protein data. The table in Supplementary file 1 gives an overview of some of the pre-trained Transformer language models available in the literature.

Using self-supervised training procedures on large databases of proteins, protein language models are able to learn very complex relationships and patterns in protein sequences through the global biome and across time.

Large protein language models, trained using one of the two approaches described above (Figure 3B) on very large databases of protein sequences, are used for downstream applications using one of two common approaches (Laskar et al., 2020). The first, called feature-based, is where a model is trained in a self-supervised manner, for instance, using one of the two approaches illustrated in Figure 3B, without any labels. The trained model’s representation of each protein sequence is then considered a feature vector for a protein that can directly be used for downstream protein prediction tasks. This is called pre-training and is what we used in the post-translational modification example in the section ‘A proof-of-principle example’ below. The second, called fine-tuning, is where a model is trained first in a self-supervised manner without any labels for the protein sequences, and then updated, or fine-tuned, using protein sequences with labels of interest. After that, the model’s fine-tuned protein sequence representations are used for downstream prediction tasks.

## Solving protein prediction tasks using transformers

Protein prediction tasks for which the Transformer has been used include predictions of protein structure, protein residue contact, protein–protein interactions (PPI), drug–target interactions (DTI), PTMs, and homology studies. The task can either be local (sites of interest within the sequence) or global (entire sequence). The fixed size Transformer representation for the local task can be obtained by taking a fixed window around the sites of interest, while the fixed size representation of a protein for a global task is achieved, for instance, by averaging the residue vectors to yield the protein sequence vector (Väth et al., 2022). It remains to be seen on which protein problems the Transformer models do not perform so well since the use of Transformer models is still spreading, and they are being used to solve more and more protein prediction tasks. Most of the state-of-the-art techniques for such predictions have been based on features from profile-to-profile comparison created from multiple sequence alignments (MSAs) of proteins using tools such as PSI-BLAST (Altschul et al., 1997) and HMMER (Finn et al., 2011). A protein profile is built by converting MSAs into a scoring system of amino acid positions based on their frequency of occurrence and is used to model protein families and domains. However, such techniques have limitations due to the existence of gaps in the protein sequences stemming from deletions and insertions (Golubchik et al., 2007) and work unsatisfactory for sequences having few to no homologs to generate MSA and profiles (Phuong et al., 2006). Moreover, predicted structural features, such as secondary structure, have also been popular when developing predictive models, but they suffer a limitation since the structural information problem is yet to be solved, which results in imperfect features (Sułkowska et al., 2012; Schmiedel and Lehner, 2019).

The results obtained using Transformer models on such tasks have been quite promising, and without the use of MSA tools that require homologous sequences, and also without structural information. A recent framework introduced by Chowdhury et al., 2022, which has a Transformer-based language model at its core, outperformed AlphaFold2 (Jumper et al., 2021), an MSA-based approach, in structure prediction for sequences that lack homologs. There are Transformer models that utilize evolutionary information extracted from MSAs during the pre-training stage, but pre-training is mostly done as a one-off process, and representation for new proteins is extracted using only the pretrained hidden states of the Transformer models. MSA tools generate alignment by searching homologs from the entire UniProt database, time-consuming (Hong et al., 2021) process, whereby generating embeddings using protein language models is less cumbersome but it also builds richer and more complete features for low homologous proteins (Wang et al., 2022).

In the following, we summarize typical problems from different fields of the life sciences, for which Transformer models have been used to aid in the prediction of protein properties. Most of these works employ pre-trained Transformer models to generate protein representations that can be used in downstream tasks for predictions.

### Structure prediction

A fundamental task that has been pursued for decades is to predict a protein’s structure. The structure is encoded in a protein’s amino acid composition, and both the composition and the structure can determine a protein’s function (Jumper et al., 2021). The protein structure prediction task can be broken down into two categories: secondary structure (α-helix, β-sheet, or coil) and tertiary structure (3D shape). These major tasks can further be broken down into other prediction tasks. For instance, predictions can be carried out to find 2D contacts, which can then be employed successively for 3D structure prediction since two residues in a sequence can be spatially close to each other in the 3D configuration (Du et al., 2021). Protein contact prediction can be formulated as either a binary classification problem (whether two residues have a close distance between their central carbon atoms), a multiclass classification problem (encapsulating real distance predictions by dividing the distance measurements into discrete bins), or as a regression problem (predicting real-valued distances). The tasks of secondary structure prediction (Elnaggar et al., 2020a; Rives et al., 2021; Brandes et al., 2021; Rao et al., 2021; Rao et al., 2019; Elnaggar et al., 2020b; Sturmfels et al., 2020) and contact prediction Rives et al., 2021; Rao et al., 2021; He et al., 2021; Sturmfels et al., 2020 have been undertaken using multiple different Transformer models, and they show great promise. For example, Rives et al., 2021 predicted secondary structure and contact by training a neural network classifier using sequence profile features combined with the representation from their ESM-1b Transformer model. They evaluated the feature combination on the Critical Assessment of protein Structure Prediction (CASP) test set (Kryshtafovych et al., 2019), and the results show an improved performance compared with other models. Other works on contact predictions include utilizing the feature combination of one-hot encoding, SPOT-1D-Single (Singh et al., 2021), and the representation from ESM-1b (Rives et al., 2021) to train a neural network classifier. This showed improvements over evolutionary-profile-based methods and over using ESM-1b representation alone (Singh et al., 2022). Moreover, a novel Transformer was pre-trained and utilized the CASP14 benchmark (Kryshtafovych et al., 2019) for contact prediction that outperformed the winner group of CASP14 contact prediction challenge (Zhang et al., 2021).

### Homology prediction

In homology prediction, a non-annotated protein with unknown biological function is characterized by finding evolutionary related sequences with known function (Gromiha et al., 2019). In microbiology and medicine, detection of remote homologs is of great interest, for instance, to detect emerging antibiotic-resistant genes (Tavares et al., 2013). The conventional approach for homology prediction has been to use MSAs, where computational tools such as MMseqs2 (Steinegger and Söding, 2017), Pfam profile (ElGebali et al., 2019), and PSI-BLAST (Altschul et al., 1997) align evolutionary related protein positions by deducing conserved sequence patterns based on evolutionary constraints that maintain the sequence’s structure and function. A major issue with these tools is that they fail to determine sequences that are distantly related (remote homology) (Wilburn and Eddy, 2020). A new method was introduced by Zare-Mirakabad et al., 2021 that utilizes a pre-trained Transformer called ProtAlbert (Elnaggar et al., 2020b) to predict a protein’s profile. To predict the profile for a protein, the protein sequence with masked tokens was fed to the model and predicted the most likely amino acids in those masked positions. The predicted profiles were compared with the sequence profiles in the HSSP dataset (Dodge et al., 1998). They concluded that the high similarity between the two profiles (predicted and HSSP database) indicates the usefulness of their approach, and that it can assist researchers in obtaining prediction profiles for new sequences. Contrastive learning, which involves finding an embedding space where similar samples are brought together while dissimilar ones pushed apart, was investigated by Heinzinger et al., 2022. The work utilized embeddings from the ProtT5 (Elnaggar et al., 2020a) pre-trained Transformer model that were mapped using a feed-forward neural network to a new embedding space. The similarity between pairs, using Euclidean distance in the embedding space, was used to find homologous sequences, as well as to identify more distant relations. They observed that this approach required significantly less protein pre-processing time compared to MSA profiles from tools such as HMMER (Finn et al., 2011). Their results not only showed similar performance to HMMER (Finn et al., 2011) profiles but outperformed it for distant relations. Their work also found that the contrastive learning approach captured structural hierarchies that provide structural similarities between proteins. Protein profile prediction without sequence alignment was undertaken by Behjati et al., 2022, who proposed a method for single protein profile prediction using the ProtAlbert (Elnaggar et al., 2020b) Transformer. Their work found that attention heads of the pre-trained Transformer captured hidden protein characteristics in the sequence, such as amino acid neighbour interaction, biochemical and biophysical amino acid properties, protein secondary structure, etc. Homology prediction has also been part of many other works to demonstrate the benefits of newly developed Transformer models (Rives et al., 2021; Brandes et al., 2021; Rao et al., 2019; Sturmfels et al., 2020).

### Mutation prediction

Mutations in proteins is another important prediction task. Mutations are a vital part in evolution and introduce diversity to protein sequences. They can either be advantageous in evolution or cause illnesses, for example, a change in a protein’s stability may cause a disease. Predicting the impact of mutations is a step towards understanding protein function and stability. The approach of pre-training and fine-tuning a Transformer network was undertaken by Yamaguchi and Saito, 2021 for mutation prediction after fine-tuning of the evolutionary information which showed better accuracy compared to using an LSTM-based approach, and by Jiang et al., 2021 to predict the pathogenic missense mutations after pre-training a Transformer and fine-tuning on paired protein sequences which outperformed a variety of existing tools. Mutation prediction was also among one of the tasks in Rives et al., 2021 and Rao et al., 2019 to verify the potential of their new pre-trained Transformer models.

### Interaction prediction

Proteins interact with other molecules, and this interaction plays an important part in cellular processes as well as in disease pathogenesis. To gain insights regarding the function of a protein in its cellular context or to develop therapeutic procedures, it is crucial to identify potential interacting molecules (McDowall et al., 2009; Dick and Green, 2018). For instance, virus proteins infect the human body through interaction with human proteins. The impact of identifying PPIs can therefore encompass vaccine design. Similarly, identifying DTI is an essential task that is critical in drug discovery. DTI prediction can contribute by narrowing the search space and prune pairs that are unlikely to bind. The field has expanded to encompass new drug discovery, repurpose drugs already in existence, and identify novel proteins that might be interaction partners for approved drugs (Öztürk et al., 2018). The existing methods of PPI and DTI are formulated as either a binary classification (interacting or non-interacting pairs), type of interaction (multiclass problem), or the strength of the interaction (regression task). Recent work in PPI has also predicted not only the interacting pairs, but also their quaternary structure (structure encompassing proteins that are closely packed together). Traditionally, PPI prediction was achieved by template-based modelling and free docking. The template-based approach involves matching sequences to related complexes for which the structure has been experimentally solved (Guerler et al., 2013) while the docking methods incorporate energy functions, and a protein’s conformation and orientation in conjunction with correlation functions from the field of pattern recognition, for instance, (Katchalski-Katzir et al., 1992) to determine the structure (Vakser, 2014). After the success of the AlphaFold (Jumper et al., 2021) model, approaches are now developed that utilize trained AlphaFold models for complex structure prediction. This is done by linking the chains of proteins and predicting the structure as if it was a single sequence (Mirdita et al., 2022; Ko and Lee, 2021). Recent works with Transformer-based models are starting to show promise in predicting interactions (Nambiar et al., 2020).

PPI was one of the tasks considered by Nambiar et al., 2020, where a Transformer model was pre-trained and fine-tuned using the HIPPIE database (Alanis-Lobato et al., 2016) they formulated it as a binary classification problem. The method surpassed the results of previously used CNN models. Lanchantin et al., 2021 proposed to predict human and novel protein interactions through multiple Transformer pre-training stages (firstly: MLM; secondly: secondary structure, contact, and remote homology prediction) and fine-tuned on virus–host PPI data (Ammari et al., 2016) for binary classification. Their approach outperformed the state-of-the-art method for this task. Xue et al., 2022 carried out cross-species PPI by pre-training a Transformer model using three separate features of proteins: sequence, structure, and function. The obtained embedding in combination with embedding from a BiLSTM model surpassed the performance when only the BiLSTM embedding was used.

For the DTI prediction, Cai et al., 2021 proposed a new protein embedding through Transformer pre-training that incorporated evolutionary information and used the model’s embeddings with a multilayer perceptron trained on several datasets (Gaulton et al., 2012; Chen et al., 2002; Chan et al., 2015; Wishart et al., 2006) to predict chemical-protein binding. The method outperformed the state of the art, which was also based on a Transformer model. A method was proposed by wang et al., 2021 to predict drug-target affinity using a regression approach by pre-training a Transformer model and using a CNN to extract features from the learned representation. They utilized multiple datasets (Tang et al., 2014; Davis et al., 2011; Liu et al., 2007) to evaluate their method. The approach proved to be more accurate than the state-of-the-art DL methods, which included a CNN model based on amino acid features and an RNN model based on protein structural features.

### Post-translational modification prediction

PTM is a process of covalent and enzymatic modification of proteins after they are synthesized (Chou, 2020). PTMs provide structural and functional diversity to proteins; however, they are also associated with major diseases like cancer. Identification of PTM sites is therefore vital for understanding it and to develop drugs for the many diseases it causes. A PTM is usually approached as a binary classification problem to identify whether a site along a protein sequence is modified or not. Prediction of lysine crotonylation, a PTM known to cause diseases like colon cancer and acute kidney injury, was undertaken by Qiao et al., 2022, where a BiLSTM network was trained on BERT (Devlin et al., 2018) embeddings of the amino acids in the protein sequences. The method outperformed the state-of-the-art model based on a CNN that utilised sequence, physicochemical properties, and Word2Vec (Mikolov et al., 2013b) features. Zhao et al., 2021 attempted to predict S-nitrosylation, a PTM that causes disorders of the cardiovascular, musculoskeletal, and nervous systems. They used representations from a pre-trained BiLSTM model and the representation from a BERT model (Devlin et al., 2018) to encode amino acids. Their approach surpassed the performance of several state-of-the-art methods, including a DL approach that had used position-specific scoring matrices (Jones, 1999) (features from MSA).

The advantage of attention mechanism is also explored in the work by Wang et al., 2020. In this work, the authors predict several PTMs, including a PTM called phosphorylation, which is one of the most studied PTMs, by including convolution layers with attention. Their framework performed better than existing methods for almost all PTMs.

## Interpreting the Transformer model

Apart from the ground-breaking performance of Transformer models, they also offer possibilities for visualization and interpretation of their attention weights. In addition to traditional approaches such as using scatter plots (Van der Maaten and Hinton, 2008; Abdi and Williams, 2010) to visualize the learned representations (Nambiar et al., 2020; Elnaggar et al., 2020a; Rives et al., 2021; Rao et al., 2019), Transformers offer other prospects for interpretation, allowing a researcher to look inside their model to better understand its function rather than using the model as a black box. The analysis of attention heads reveal the weight assignments between pairs of input tokens, and these weights can be used to draw conclusions about the interactions between tokens that contributed to a model’s decision (Hao et al., 2021). Note, however, that such an analysis does not necessarily point out the feature importance in the representation of the Transformer. Examples of some commonly used Transformer model visualizations include assessments of both the attention mechanism and the embeddings using, for example, attention weights (Elnaggar et al., 2020a) and heatmaps (Brandes et al., 2021; Zhang et al., 2021; Yamaguchi and Saito, 2021; Vig et al., 2020). Attention weight visualization allows the portrayal of attention weights between an amino acid to the other amino acids in the form of intensity of line connections. Heatmaps can be used to show the colour shades for the different attention heads across the different layers for each amino acid, amino acid to amino acid maps using averaged weights of the heads across the network layers, etc. Transformer visualizations are not limited to the only ones listed here as new techniques are continually suggested in scientific publications which shows how versatile Transformer models are (Chefer et al., 2021). Moreover, the intricacy in the existing ways of interpreting Transformer models, especially its multihead attention, is also being improved so that model’s internal learnings can be made more easy for analysis (Hao et al., 2021; Vig, 2019b).

**Figure 5.**
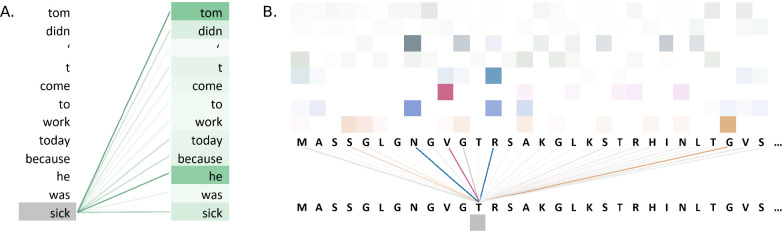
Visualisations of the attention weights in transformer models. (**A**) A visualization of the attention weights in a BERT model. The weights are from the first attention head of the eighth layer of the model. The model has a total of 12 layers and 12 attention heads. In this example, the model connects the words 'tom' and 'he' to the word 'sick' (darker lines indicate larger weights). Visualization inspired by BertViz (Vig, 2019a; https://github.com/jessevig/bertviz; Vig, 2022). (**B**) Attention weights visualization showing that a protein language model learned to put more weight from one residue onto four other residues in one layer. The shades of a particular colour (horizontal order) correspond to an attention head in the layer of the Transformer. Dark shades indicate stronger attention and are hence shown with darker lines connecting the tokens.

Figure 5A illustrates attention weights in an example using the BERT model. The model identifies dependencies in the sentence by showing which words attend to the word 'sick' in an attention head of a layer of the model. The model clearly connects the words 'tom' and 'he' to the word 'sick', indicating that the model has learnt to identify context in the sentence.

Attention weight visualization was utilized by Elnaggar et al., 2020a, who visualized the attention weights of each amino acid residue onto the other residues in the protein sequence, where a darker line represented a higher attention weight, as in Figure 5B. Specifically, they analysed the residue contacts (protein structural motifs) crucial for zinc-binding and found that the model had learned to put more weight from one residue onto three other residues in one layer, and all these residues collectively were involved in the ground truth binding coordination. Heatmaps were used by Yamaguchi and Saito, 2021, who analysed effects of fine-tuning Transformer model with the use of evolutionary properties of proteins. The pre-trained and fine-tuned maps of a protein from the final layer were compared to the contact maps computed from its tertiary structure. It was observed that the fine-tuned model’s maps resembled that of contact maps and this pattern was absent from the map prior to fine-tuning. The visualization indicated that the structural information was captured by the model after fine-tuning on evolutionary related sequences. Heatmaps were also used by Zhang et al., 2021, where they proposed an evolutionary information-based attention (co-evolution attention). They visualized the maps with and without the evolutionary information-based attention and concluded that their attention component was effective in extracting contact patterns.

Such visualizations can thus be used to understand biological aspects of different protein properties, and by visualizing a Transformer model’s internal state we can gather and present deeper biological insights.

## Transformer language model adoption

**Figure 6.**
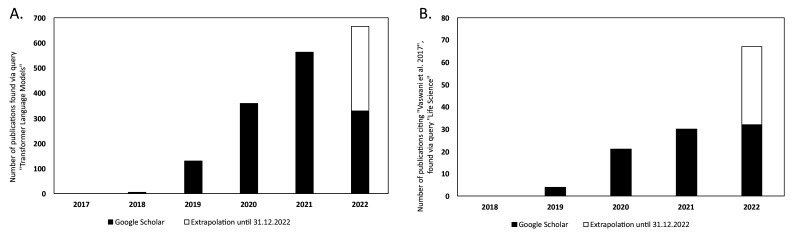
Yearly number of publications on Google Scholar for the years 2017–2021 and extrapolated count for the year 2022. For: (**A**) the search query 'Transformer Language Model' and (**B**) the search query 'Life Science' that have cited the original Transformer paper by Vaswani et al., 2017.

There has been a steady increase in the volume of scientific publications relating to Transformer-based models since their introduction in 2017 (Vaswani et al., 2017). This is evident in the progresses of both NLP and in computational biology and bioinformatics research. Figure 6A illustrates the yearly count of publications from 2017 to 2021 for the query 'Transformer Language Model' in Google Scholar, 2022. This resulted in a total number of publications of 1388. The plot has been extended to include the year 2022 by extrapolating the counts for 2022 (counts of scientific publications were until 2022-07-01 at the time of writing this article) until the end of the year. There is clearly an increase in how often Transformer-type of models are mentioned in the literature.

**Figure 7.**
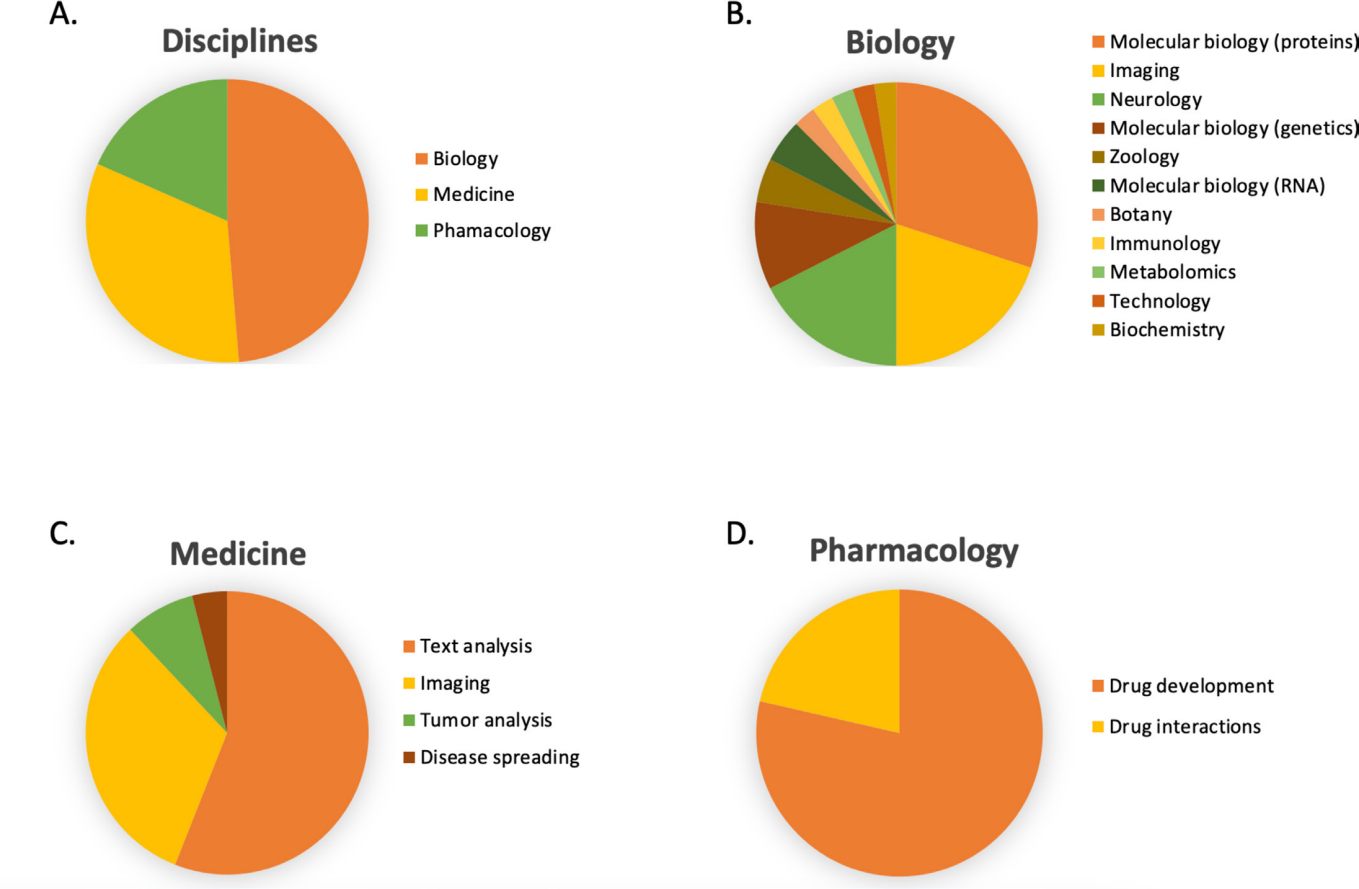
The article counts for the three main disciplines (medicine, pharmacology, and biology) and percentage breakdown of their sub-categories in Google Scholar citing the 'Attention is all you need' paper by Vaswani et al., 2017. The search was based on the query 'Life Science' and included all scientific research papers from 2017 to 2022 (cut-off on 2022-06-23).

In Figure 6B, the number of publications per year is illustrated for scientific publications related to Transformer-based models which were identified by searching within articles in Google Scholar citing the 'Attention is all you need' paper by Vaswani et al., 2017. The search was based on the query 'Life Science' and included all scientific research papers from 2017 to 2022 (cut-off on 2022-06-23). We excluded review papers and theses from the analysis. Articles focusing solely on method developments were excluded from the query results as well, leaving us with a total of 87 publications. The results were sorted by three main disciplines: medicine, pharmacology, and biology, as shown in Figure 7. Within the main disciplines, articles were sorted by their sub-categories. The increased use of Transformer models in the different areas of bioinformatics indicates that it is an effective model to use when studying different protein properties.

## A proof-of-principle example

To illustrate how the features learned by the Transformer model can be used to directly improve results over traditional protein features, we have conducted a pilot study in phosphoglycerylation prediction. Phosphoglycerylation is a type of PTM discovered in human cells and mouse liver which occurs when the amino acid lysine in a protein sequence is covalently modified by a primary glycolytic intermediate (1,3-BPG) to form 3-phosphoglyceryl-lysine. This PTM has been found to be associated with cardiovascular diseases like heart failure (Bulcun et al., 2012).

The task was to predict phosphoglycerylation, and for this we used the dataset from Chandra et al., 2019 which was originally obtained from the Protein Lysine Modification Database (PLMD, available at http://plmd.biocuckoo.org). The features used by Chandra et al., 2019 were based on position-specific scoring matrices that were obtained using the PSI-BLAST toolbox (Altschul et al., 1997), which is an MSA tool, and then they calculated its profile bigrams (Sharma et al., 2013) (a type of feature extraction) to produce the final feature set. We used this feature set, denoted *BigramPGK*, and compared the results to results when using features extracted from two pre-trained Transformer models. Additionally, we also used a second baseline feature set that composed of 10 commonly used physicochemical/biochemical properties of each amino acid (Yu et al., 2017; Chen et al., 2020; Cortés and Aguilar-Ruiz, 2011; Liu et al., 2012). We denote this feature set as Phy + Bio. The features were length of side chain, molecular weight, free energy of solution in water, melting point, hydrostatic pressure asymmetry index, isoelectric point, hydrophobicity index, ionization equilibrium constant (pK-a), pK (-COOH), and net charge. The Transformer models were the ESM-1b (Rives et al., 2021) and the ProtT5-XL-UniRef50 (Elnaggar et al., 2020a). We used two aggregation techniques for the ESM-1b model since it has a restriction on the length of the protein sequence that can be processed. First, the protein sequence was split into multiple parts by using 878 consecutive amino acids at a time, starting at each amino acid in the sequence. The ESM-1b model was used to extract a feature vector for each such subsequence of length 878, and the feature vectors were averaged to obtain a single feature vector for the entire protein sequence. We denote this approach *ESM1b-avg*. The second approach was to again split the protein sequence up into subparts of length 878, but this time splitting from where the last split ended and finally concatenating to get the resulting feature vector of the protein sequence. We denote this approach *ESM1b-concate*. We denote the features extracted from the ProtT5-XL-UniRef50 model as *T5*, and this model accepts variable-length input sequences so there was no need to aggregate multiple feature vectors manually.

After obtaining the representation of the protein sequences from the Transformer models, the feature of each sample was extracted by examining the sites of interest in the protein sequences and selecting the window size around those sites. The samples were extracted based on the standard practices, as outlined, for example, by Ramazi and Zahiri, 2021, which include to consider a window size of 15 amino acid residues upstream and 15 amino acid residues downstream of the lysine sites (López et al., 2017; Jia et al., 2016; Xu et al., 2018), to disregard the sites which did not have enough residues to make up the full upstream and downstream window (Wang et al., 2017; Saethang et al., 2016), and to take unlabelled sites as non-phosphoglycerylated samples only if the protein has two or more confirmed PTM sites in its sequence (Khalili et al., 2022; Trost and Kusalik, 2013). These conditions were applied to all the feature sets. The resultant dataset had a total of 526 samples (relating to each lysine) containing 101 phosphoglycerylated samples (positive labels) and 425 non-phosphoglycerylated samples (negative labels). We used random under-sampling to resolve the class imbalance ratio from 1:4 to 1:1.5 by randomly selecting the negative labels (Ramazi and Zahiri, 2021). The final number of samples used in the experiment was 253 (with 101 phosphoglycerylated and 152 non-phosphoglycerylated samples).

We used and compared five classifiers: logistic regression with ridge regularization (denoted *LR*), a support vector machine with a polynomial kernel (denoted *SVM (poly*)), a support vector machine with a radial basis function kernel (denoted *SVM (RBF*)), random forest (denoted *RF*), and finally a light gradient-boosting machine (denoted *LightGBM*). We set aside 51 samples for final test of each model (maintaining the same ratio between the positive and negative labels, i.e., 1:1.5) and performed fivefold cross-validation on the remaining 202 samples to select the models’ hyper-parameters with standard scaling of the data, based on the training set in each cross-validation round. The hyper-parameters were tuned using Hyperopt (Bergstra et al., 2013) with 15 evaluations.

We thus had five datasets (Phy + Bio, BigramPGK, T5, ESM1b-avg, and ESM1b-concate) and five classification models (LR, SVM (poly), SVM (RBF), RF, and LightGBM). We evaluated each model on all datasets using accuracy (ACC) and the area under the receiver operating characteristic curve (AUC), reporting both the fivefold cross-validation (CV) scores (those used to select the hyper-parameters) and the score obtained on the held-out test set (the 51 set-aside samples mentioned above). The results are presented in Table 1.

**Table 1.** The performance on five datasets, i.e. the five feature sets (Phy + Bio, BigramPGK, T5, ESM1b-avg, and ESM1b-concate) by five classification models (LR, SVM (poly), SVM (RBF), RF, and LightGBM) evaluated using accuracy (ACC) and the area under the receiver operating characteristic curve (AUC). The reported cross-validation results are the mean over the five CV rounds. Standard errors for both CV and test are in the parenthesis. The highest scores are highlighted in bold. CV: five-fold cross-validation; Test: held-out test set.

		LR		SVM (poly)		SVM (RBF)		RF		LightGBM	
		ACC	AUC	ACC	AUC	ACC	AUC	ACC	AUC	ACC	AUC
Phy +Bio	CV	0.550 (0.032)	0.546 (0.012)	0.614 (0.017)	0.550 (0.027)	0.545 (0.035)	0.552 (0.013)	0.609 (0.010)	0.564 (0.034)	0.525 (0.027)	0.498 (0.029)
	Test	0.471 (0.071)	0.395 (0.083)	0.588 (0.070)	0.552 (0.083)	0.471 (0.071)	0.371 (0.082)	0.628 (0.068)	0.489 (0.084)	0.529 (0.071)	0.503 (0.084)
BigramPGK	CV	0.678 (0.026)	0.686 (0.019)	0.590 (0.025)	0.723 (0.025)	0.599 (0.004)	0.711 (0.030)	**0.698** (**0.008**)	0.707 (0.025)	0.629 (0.024)	0.627 (0.028)
	Test	0.628 (0.068)	0.686 (0.074)	0.647 (0.068)	0.666 (0.076)	0.608 (0.069)	0.668 (0.076)	**0.686** (**0.066**)	0.742 (0.069)	**0.706** (**0.064**)	0.742 (0.069)
T5	CV	0.704 (0.038)	0.742 (0.039)	0.713 (0.035)	0.744 (0.038)	0.713 (0.034)	0.737 (0.041)	0.634 (0.021)	**0.747** (**0.041**)	0.668 (0.022)	**0.756** (**0.018**)
	Test	0.647 (0.068)	0.726 (0.070)	0.628 (0.068)	0.726 (0.070)	0.628 (0.068)	0.737 (0.069)	0.647 (0.068)	0.736 (0.070)	0.471 (0.071)	0.592 (0.081)
ESM-1b-avg	CV	0.768 (0.025)	0.830 (0.025)	0.748 (0.022)	0.826 (0.028)	0.599 (0.004)	0.785 (0.055)	0.639 (0.015)	0.745 (0.058)	0.708 (0.020)	0.741 (0.044)
	Test	0.726 (0.063)	**0.803** (**0.061**)	0.667 (0.067)	0.813 (0.059)	0.608 (0.069)	**0.811** (**0.060**)	0.628 (0.068)	0.719 (0.071)	0.647 (0.068)	**0.748** (**0.068**)
ESM-1b-concate	CV	**0.782** (**0.012**)	**0.852** (**0.015**)	**0.792** (**0.014**)	**0.853** (**0.015**)	**0.773** (**0.015**)	**0.844** (**0.023**)	0.609 (0.017)	0.742 (0.048)	**0.718** (**0.017**)	0.755 (0.039)
	Test	**0.745** (**0.062**)	0.797 (0.062)	**0.745** (**0.062**)	**0.824** (**0.057**)	**0.726** (**0.063**)	0.798 (0.061)	0.628 (0.068)	**0.850** (**0.053**)	0.667 (0.067)	0.726 (0.070)

The Transformer models perform better in general than the BigramPGK protein features (based on MSAs) and the Phy + Bio features on the accuracy and AUC metrics across all the classifiers, except for the RF classifier where BigramPGK attained the highest accuracy on the fivefold cross-validation. Out of the five features, Phy + Bio had the lowest performance. We see that the concatenated features from the ESM-1b Transformer model generally perform better than all of the other feature sets, including the Transformer features (averaged features from ESM-1b, and the T5 features). While the differences are not always significant, it is clear that the trend is that the Transformer features perform better.

## Outlook

The Transformer family of models has shown large improvements over RNNs and other DL-based models. In just a few years, they have been used for many different prediction tasks and their representations have been used with very promising results. In contrast, it took decades for conventional features based on MSAs to reach their current performances. The Transformer models have their own set of limitations, and future improvements in their architecture will likely give further boosts in their performance.

For instance, the standard attention mechanisms can only process fixed-length input sequences. For longer sequences, they need to be split into smaller fragments before being fed to a model. However, splitting a sequence up means context is being lost beyond the split boundary. Recent developments have attempted to overcome the fixed-length issue, where, for instance, some variants allow hidden states from previous fragments to be used as inputs for the current fragment (Elnaggar et al., 2020a; Dai et al., 2019). ProtT5-XL-UniRef50 model used in the section ‘A proof-of-principle example’ uses the same technique to pass information from one fragment to the other in the protein sequence. This allows a Transformer model to consider very long dependencies and at least in theory handle unlimited-length contexts since the information from one segment can be passed on to the next infinitely (Wang et al., 2019). Furthermore, some transformer models need the users to pre-process the sequences to adhere to a sequence length limit. This was apparent with the ESM-1b model in the ‘A proof-of-principle example’. The workaround was to break the longer sequences into fragments (maximum lengths of 878 in this work) to get the Transformer representations, which was then concatenated to produce a representation for the entire sequence. That approach worked out as the best-performing features in this study out of the features compared. Fragmenting the sequence of course results in loss of some contexts, and future improvements to the sequence length limit can lead to more robust performances.

The attention mechanism, which is an integral part of Transformer models, also brings a limitation when it comes to long sequences. Since each token attends to every other token, the memory and computational complexity of the model increases quadratically in the attention layers with respect to the sequence length. A solution using sparse attention mechanism was proposed by Zaheer et al., 2020 that changed the complexity from quadratic to linear and allowed up to eight times longer sequences to be handled on similar hardware. Their proposed attention mechanism consisted of three parts: (1) making some tokens global which attend to the entire sequence, (2) all tokens attend to a set of local neighbouring tokens, and (3) all tokens attend to a set of random tokens. This technique also allows the Transformer to handle longer contexts. Moreover, the memory requirements and the complexity of Transformer models was also addressed by Kitaev et al., 2020, who introduced two techniques: (1) they replaced the dot-product attention with a locality-sensitive hashing which deals with a subset of nearest neighbours in high-dimensional spaces for the attention computation which saw the reduction of complexity from quadratic to log linear, and (2) they utilized reversible residual layers in place of standard residuals, thereby allowing the storage of activations only once instead of in every layer, which makes it much more memory efficient. Furthermore, Sourkov, 2018 proposed to replace pairwise dot-product attention mechanism with an IGLOO-base block to a get computational advantage. This new block did not require the computation of the full self-attention matrix, but rather a constant number of elements from distant parts of the sequence. This is particularly useful for bioinformatics tasks since these tasks often comprise long sequences.

The Transformer models, even though emerging as the new workhorse for NLP, were found not to perform well in comparison to LSTM in some tasks. For instance, Tran et al., 2018 compared LSTMs and Transformers in their ability to model the hierarchical structure in sentences. The tasks that were performed were subject–verb agreement and logical inference. They observed that the LSTMs consistently outperformed Transformers and the performance gap increased with the distance between the subject and the verb in a sentence. The task of logical inference, which is to predict logical relations between pairs of sentences, was also found to be modelled better with the LSTM architecture, especially in longer sequences. Work by Hahn, 2020 also showed that Transformers had problems to accurately evaluate logical formulas and to model hierarchical structures. Moreover, regarding linguistics, a Transformer model called GPT-2 had problems to learn poetry and rhyming (Wang et al., 2021). Its successor, called GTP-3, did a bit better on this task, but not as much improvement as was seen in tasks like arithmetic. These shortcomings of the Transformer models are important to be aware of since they could also be considerable factors in protein prediction tasks for which these or similar properties are critical.

A better performance on downstream task can usually be achieved by increasing the Transformer model’s size (adding more layers and more parameters). Such high-capacity models face both memory limitation and longer training times. Lan et al., 2019 managed to limit these issues by employing techniques in their framework that lowers the memory consumption and increases training speed. These include projecting the word embeddings into a lower dimensional embedding, thereby resulting in parameter reduction, parameter sharing in feed-forward network and attention across the layers to improve parameter efficiency and employed a sentence prediction loss that helped improve downstream task performance.

In the MLM approach, where some of the tokens are masked, the model neglects dependency between these masked positions. One way of overcoming this limitation is to utilize the benefits of autoregressive language modelling and to combine it with bidirectional context capturing used in MLM, instead of the original MLM (Yang et al., 2019). Moreover, the standard MLM approach is computationally expensive because it learns from only about 15% of the tokens at a time in most models (Wettig et al., 2022). In recent developments, Clark et al., 2020 proposed a sample-efficient pre-training method called *replaced token detection* that allows the model to learn from all the input tokens, unlike the masked subset approach in MLM.

The recent improvements to the Transformer model architectures indicate that this model class is still in its infancy, is clearly under fast development, and shows much promise as the architecture continues to enhance and expand. Many developments are coming in from multiple directions, such as from ML in general, from NLP, from computer vision, and from computational biology and bioinformatics, among other areas. These developments are crucial since the original Transformer architecture was designed and optimized for natural language tasks; the application of these models to biological data such as protein sequences, which are usually longer, has the possibility of running into high computational costs and memory limitation as well as suboptimally capturing very long-range dependencies. We can expect many more improvements in the years to come, and we can suppose that whatever limitations exist today will be addressed tomorrow.

A trend in the development of large Transformer models in NLP has been to build larger and larger models. 'Standard' models in NLP today have hundreds of billions of model parameters, such as Openai’s *GPT-3* model with 175 billion model parameters (Brown et al., 2020) or Microsoft and Nvidia’s *Megatron-Turing NLG* model with 530 billion model parameters (Smith et al., 2022), but the very latest models have over a trillion model parameters (Fedus et al., 2021; Narayanan et al., 2021). This trend with ever larger models is unlikely to be sustainable since they require enormous amounts of memory and compute resources, and therefore severely limit who can build and train such models. But the trend is nevertheless clear that larger and larger models are built and are more successful. These models are also trained on ever larger sets of data. We can expect both trends to follow into computational biology and bioinformatics, with larger models trained on larger sets of data. Such a trend might limit future protein research to resource rich research institutes and companies and prevent such research to be performed at universities with limited resources.

### Conclusions

This work has reviewed the potential of the Transformer models for protein prediction tasks. It has analysed some of the issues faced by the existing deep learning models and described how the latest language models, based on the Transformer, are proving to be promising models for protein prediction tasks. Transformer-based models are producing state-of-the-art results on many diverse tasks. This indicates that they are very capable models able to find relevant, important, and general features in and relationships between amino acid residues in a protein sequence. Transformer models can be analysed through their attention weights and an interpretation of the model internals can give more insight into the prediction task, and even lead to new knowledge about the underlying biology. As for all ML models, there are shortcomings also with the Transformer model, such as the quadratic growth in the memory requirement and the computational complexity of the attention layers as functions of the sequence length, the fact that the attention mechanisms process fixed length input sequences, the extensive pre-training which leads to longer training time for larger models, inadequacies in the MLM pre-training procedure, etc. Despite these shortcomings, the performance of Transformer models has been attracting a much interest and efforts from the ML community to improve the models as much as possible in the respective fields. While the Transformer model has been the go-to model in NLP tasks since 2017, their capabilities are just beginning to be explored when it comes to modelling proteins for different prediction tasks. Furthermore, it could be that Transformer models alone may not be the best approach for all the protein prediction tasks and that other or traditional methods would be required, perhaps in combination with components from Transformers, to obtain results past the current state-of-the-art methods. It is also important to be aware of other differences between Transformers and other methods, and that, for instance, differences in the training procedures, or other aspects of the whole analysis pipeline, could at least in part be the reason for some of the recent improvements. For example, the MLM pre-training and finetuning procedure has also been used with CNN models, and has shown promising results (Yang et al., 2022). The AlphaFold model uses attention mechanism from Transformers to extract information in MSAs that shows Transformer model component with traditional features work quite well. Moreover, the breakthrough performance of the Transformer models has inspired other deep learning models to incorporate similar architectural enhancements. It will be interesting to follow the developments in Transformer-based models and other deep learning models as a whole and its application to understanding proteins and its properties.

We hope the discussion in this review provides the readers, both those experienced in and those without experience in ML, with a general understanding of DL and specifically about how the Transformer model from NLP is adopted to predict properties of proteins. Specifically, the proof-of principle example shows how the Transformer models can be used as general feature extractors that can improve results compared to traditional protein features, such as those based on MSAs. The result, however, does not prove that Transformer model representations are better in general compared, for instance, to MSAs but does show that this is a promising avenue to be explored further since there are recurrent evolutionary relations captured in the representations from such type of language models (Bepler and Berger, 2021). In the example, we used standard models and used them as they were, but the future of computational biology and bioinformatics likely contains special-purpose models and model and training developments specifically made for analysing protein data that further improve such results. These are exciting times to follow the developments in the fields of computational biology and bioinformatics, which will likely be heavily based on Transformer models for the foreseeable future.

## Additional files

Supplementary file 1. Some of the commonly used pre-trained Transformer models in the literature. The higher the number of Transformer parameters, the larger the model.
