# Supplementary material for: Transformer-based deep learning for predicting protein properties in the life sciences
Source: eLife. 2023 Jan 18;12:e82819. doi: 10.7554/eLife.82819 (PMC9848389; doi:10.7554/eLife.82819)
Supplement: Supplementary file 1. — The higher the number of Transformer parameters, the larger the model. [file elife-82819-supp1.docx]

Supplementary file 1: Some of the commonly used pre-trained Transformer models in the literature. The higher the number of Transformer parameters, the larger the model.

| Work | No. of Parameters | Database (no. of proteins) for pre-training | Link |
| --- | --- | --- | --- |
| TAPE (67) | 38M | Pfam database (31M) | https://github.com/songlab-cal/tape |
| ESM-1b (41) | 652.4M | UniParc (250M) | https://github.com/facebookresearch/esm |
| Protein-BERT (42) | 16M | UniRef90 (~106M) | https://github.com/nadavbra/protein_bert |
| ProTrans (40, 68) | ProtBert – 420M | UniRef100 (216M), BFD (2.1B) | https://github.com/agemagician/ProtTrans |
|  | ProtAlbert – 224M | UniRef100 (216M), BFD (2.1B) |  |
|  | ProtXLNet – 409M | UniRef100 (216M) |  |
|  | ProtTXL – 567M, ProtTXL-BFD – 562M | UniRef100 (216M)  BFD (2.1B) |  |
|  | ProtElectra – 420M | UniRef100 (216M) |  |
|  | ProtT5-XL – 3B  ProtT5-XXL – 11B | UniRef50 (49M) |  |
|  | ProtT5-XL – 3B  ProtT5-XXL – 11B | BFD (2.1B) |  |
| MSA Transformer (43) | 100M | UniRef50 (26M MSAs) | https://github.com/facebookresearch/esm |
| PRoBERTa (69) | 44M | UniProtKB/SwissProt (450K) | https://github.com/annambiar/PRoBERTa |
